# *Trematocranus pachychilus*, a new endemic cichlid from Lake Malawi (Teleostei, Cichlidae)

**DOI:** 10.3897/zookeys.743.22814

**Published:** 2018-03-14

**Authors:** Katrien Dierickx, Mark Hanssens, Bosco Rusuwa, Jos Snoeks

**Affiliations:** 1 Ichthyology, Section Vertebrates, Department Biology, Royal Museum for Central Africa, Tervuursesteenweg 13, B-3080 Tervuren, Belgium; 2 Department of Biology, Chancellor College, University of Malawi, Zomba, Malawi; 3 Laboratory of Biodiversity and evolutionary genomics, Leuven University (KU Leuven), Charles Deberiotstraat 32, B-3000 Leuven, Belgium

**Keywords:** Cichlid, Lake Malawi, new species, thick lips, *Trematocranus pachychilus* sp. n.

## Abstract

A new species of *Trematocranus*, *T. pachychilus* **sp. n.**, is described from Lake Malawi. So far, it has only been found at Jafua Bay, Mozambique. It can easily be distinguished from *T. labifer* by its molariform pharyngeal dentition. A morphometric study, including 24 measurements and 15 counts, was done to compare the new species with *T. microstoma* and *T. placodon*. *Trematocranus pachychilus* is characterised by its thick lips. This species further differs from *T. microstoma* by its bicuspid (vs. unicuspid) outer oral teeth, wide (vs. small) pharyngeal bone, and its head shape. It resembles *T. placodon*, from which it can be distinguished by its straight to concave head profile (vs. rounded), less-developed pharyngeal bones (vs. hypertrophied), and the presence of small to minute teeth on the lateral parts of the dentigerous area on the lower pharyngeal bone. A key to the species of *Trematocranus* is provided.

## Introduction

Lake Malawi, like most other African Rift lakes, is home to a large number of endemic cichlids, that in each lake radiated into many genera and species in a very short period. Some sources estimate that there are more than 800 species of cichlids in Lake Malawi alone (e.g., Snoeks 2000). Because of the explosive radiation, many species and genera complexes still need to be taxonomically resolved.

In Lake Malawi, haplochromines constitute the major part of the cichlids. The endemic genus *Trematocranus* Trewavas, 1935, currently includes three valid species: *T. microstoma* Trewavas, 1935, *T. labifer* (Trewavas, 1935), and *T. placodon* (Regan, 1922) (Eccles and Trewavas 1989). These fish can grow to sizes of 250 mm TL (Maréchal 1991). The genus is characterised by a typical melanin pattern with large supra-pectoral and supra-anal spots situated on the upper lateral line, extending to the base of the dorsal fin and usually overlaid on fainter bars. There is also an opercular spot and one at the end of the caudal peduncle (Eccles and Trewavas 1989). The teeth are placed in 3 - 8 inner teeth series in the lower oral jaw and the outer teeth are long and recurved, either bicuspid or simple (Eccles and Trewavas 1989).

*Trematocranus microstoma* is the type species of the genus. It has simple, long, and slender teeth in the oral jaws. They are recurved and placed in 6 - 8 inner teeth series anteriorly (Eccles and Trewavas 1989). The pharyngeal teeth are considerably enlarged and molariform (Snoeks and Hanssens 2004). *Trematocranus labifer* has bicuspid, or simple anterior, oral teeth in three or four series. The teeth on the lower pharyngeal bone are all compressed, sharp, and not enlarged or molariform (Eccles and Trewavas 1989, Snoeks and Hanssens 2004). The third species, *T. placodon*, has bicuspid, or simple anterior, oral teeth in three or four inner teeth series. The lower pharyngeal bones are massively enlarged with large, flattened, and molariform teeth (Eccles and Trewavas 1989, Snoeks and Hanssens 2004). This adaptation is clearly related to a diet of snails (Mtethiwa et al. 2015).

Although Eccles and Trewavas (1989) and Snoeks and Hanssens (2004) limited the genus to these three large species, six more species have been placed in the genus at some point during their taxonomic history. *Trematocranus auditor* Trewavas, 1935, *T. brevirostris* Trewavas, 1935, *T. jacobfreibergi* Johnson, 1974, and *T. trematocephala* (Boulenger, 1901) are now assigned to the genus *Aulonocara* (Eccles and Trewavas 1989, Maréchal 1991). Eccles and Trewavas (1989) placed *T. brevirostris* in *Aulonocara*. Snoeks and Hanssens (2004) proposed to assign it to *Aulonocara* or *Otopharynx*, since its sensory pores are less developed than in most other *Aulonocara* species. The species is still mentioned as *T. brevirostris* in a popular account of Lake Malawi cichlids by Konings (2016). *Trematocranus peterdaviesi* Burgess and Axelrod, 1973, is now in the genus *Alticorpus*, and *T. intermedius* (Trewavas, 1935) in *Tramitichromis* (Eccles and Trewavas 1989).

Already in 2004, Snoeks and Hanssens mentioned the possibility of a fourth species of *Trematocranus*, then known as *T.* sp. ‘thicklip-bicuspid’, based on the oral dentition and the hypertrophied lips. These specimens were all collected at Jafua Bay, north of Cobue, and near the island of Likoma, Mozambique, in 1998. In this bay, *T. placodon*, which has a wider distribution, is also found (Snoeks and Hanssens 2004). So far, the new species of *Trematocranus* has not been found in another location.

Below, the species new to science as *Trematocranus pachychilus* sp. n. will be proposed.

## Materials and methods

In total, 30 specimens of *Trematocranus* of the collection at the RMCA (Royal Museum of Central Africa, Tervuren) were examined: seven *T. pachychilus* sp. n., 13 *T. placodon*, and ten *T. microstoma*. All specimens used in this study were collected by the taxonomy team of the SADC/GEF Lake Malawi/Nyasa/Niassa project during several expeditions on Lake Malawi in 1997 and 1998. They were all caught by bottom trawling using the RV Usipa.

The measurements and counts performed here follow Snoeks (2004). On the lower pharyngeal bone, one additional measurement was taken. The pharyngeal depth was measured from the anteriormost part of the dentigerous area to the indentation between the central area and the rostral keel on the ventral side of the bone. In total, 24 measurements, 16 counts (including the number of vertebrae via X-rays), and some observations on the body, head shape, and the colour pattern in alcohol were made. The abbreviations of these measurements and counts are given in Table 1.

**Table 1.** Measurements and meristics for *Trematocranus pachychilus* sp. n. (holotype and six paratypes), compared with the ranges and means of the specimens of *T. microstoma* and *T. placodon*. Key: * only nine specimens used, ** only four specimens used, *** only six specimens used).

	*T. pachychilus* sp. n.		*T. microstoma*	*T. placodon*
	Holotype	Type series		
		Range, mean (n = 7)	Range, mean (n = 10)	Range, mean (n = 13)
Morphometrics				
Standard length (SL) in mm	154.6	117.7–154.6 (139.0)	126.7–182.7 (149.86)	116.0–163.1 (141.8)
**As % SL**:				
Body depth (BD)	39.9	36.0–41.8 (38.4)	35.7–41.6 (39.3)	38.0–41.9 (39.8)
Head length (HL)	32.0	31.5–33.8 (32.2)	31.6–34.4 (32.4)	30.0–34.3 (32.5)
Prepectoral distance (PrP)	31.0	30.1–34.3 (32.1)	31.1–34.4 (32.3)	29.9–34.1 (32.2)
Predorsal distance (PrD)	36.5	34.8–37.7 (36.2)	34.7–40.4 (37.0)	35.0–39.2 (37.4)
Preventral distance (PrV)	37.8	36.9–41.5 (38.4)	37.5–44.0 (40.1)	36.2–40.3 (38.4)
Preanal distance (PrA)	65.9	64.0–67.0 (65.5)	63.7–70.5 (65.7)	61.3–67.9 (64.4)
Dorsal fin base (DFB)	56.9	54.7–59.4 (56.9)	55.2–58.9 (57.4)	54.1–60.0 (56.1)
Anal fin base (AFB)	19.5	19.1–20.8 (20.0)	20.1–22.6 (21.3)	17.7–22.2 (20.0)
Caudal peduncle length (CPL)	20.0	19.2–20.0 (19.6)	16.8–21.4 (18.8)	18.2–20.8 (19.6)
Caudal peduncle depth (CPD)	13.3	12.2–13.5 (13.0)	12.5–14.5 (13.3)	12.3–14.3 (13.2)
**As % HL** :				
Head width (HW)	46.4	42.7–47.6 (45.7)	43.1–46.5 (44.8)	43.8–49.9 (46.5)
Premaxillary processus length (PPL)	26.4	26.3–31.5 (29.2)	27.1–33.1 (29.5)	26.8–33.3 (29.5)
Snout length (SnL)	37.6	34.8–40.9 (37.4)	38.0–42.4 (40.3)	35.7–41.6 (37.6)
Lachrymal depth (LaD)	26.9	22.9–29.7 (26.9)	29.3–32.0 (30.5)	25.6–29.5 (27.4)
Cheek depth (ChD)	25.9	22.2–27.3 (24.6)	23.8–28.1 (26.8)	24.0–29.5 (26.6)
Eye diameter (ED)	26.4	25.2–29.5 (27.5)	21.2–25.5 (23.4)	23.1–27.1 (24.7)
Interorbital width (IOW)	28.3	24.7–29.8 (27.3)	25.1–29.3 (27.6)	25.5–31.3 (28.6)
Lower jaw length (LJL)	28.4	28.3–30.0 (29.0)	26.2–30.2 (28.2)	26.2–32.4 (29.0)
Lower pharyngeal depth (PHD)	9.7	9.4–10.4 (10.0)	8.0–10.8 (9.1)	13.8–16.8 (15.5)
Length lower ph. bone (LPHL)	28.7	21.8–30.8 (27.6)	20.1–30.0 (27.2)	29.8–35.9 (33.2)
Width lower ph. bone (LPHW)	33.2	29.7–33.2 (31.4)	19.1–30.9 (28.1)	36.1–48.7 (39.1)
Lower ph. dent. area length (DEAL)	19.4	18.4–20.1 (19.3)	16.1–21.3 (18.3)	21.6–24.3 (23.0)
Lower ph. dent. area width (EAW)	24.4	21.9–24.4 (23.0)	18.8–23.6 (18.3)	24.2–27.5 (25.5)
Meristics				
Longitudinal series	33	32–35	32–35	30–34
Upper lateral line	22	22–24	21–23	19–22
Lower lateral line	14	14–18	13–19	14–18
Upper gill rakers	4	3–4	3–4	3–5
Lower gill rakers	9	8–9	8–10	7–9
Dorsal fin spines	15	15–16	15–16	15–16
Dorsal fin rays	11	11–12	11	9–11
Anal fin rays	8	8–10	8–11	8–10
Pectoral fin rays	15	14–15	13–15	14–15
Upper oral jaw teeth	40	36–47	39–47 *	41–57
Lower pharyngeal jaw teeth rows	24	23–27	24–29	11–23
Vertebrae	31	31–32	31–32 **	31–32 ***

Principal component analysis (PCA) was performed in R to explore the multivariate data set. Measurements were log-transformed and the correlation matrix used. When using log-transformed measurements, the individual loads of all variables on the first principal component (PC 1) are of the same magnitude and sign and PC 1 can therefore be regarded as a proxy for multivariate size (Jolicoeur 1963, Snoeks 2004, Van Steenberge et al. 2015). The correlation matrix was used for the raw meristic data. The number of vertebrae and anal and pelvic fin spines and rays were all without variation and thus were excluded from the analysis.

## Results

### Comparative morphometrics

A general PCA on the 24 log-transformed measurements including all specimens did not show a clear separation between the three species (figure not shown). In subsequent comparative analyses of *T. pachychilus* with *T. placodon* and *T. microstoma* separately, the new species was found clearly distinct from the two others.

**Figure 1.**
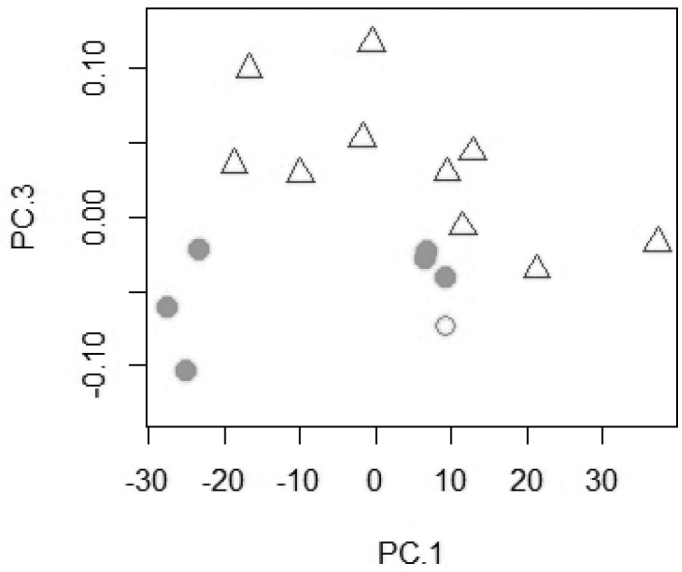
Scatter plot of PC 1 against PC 3 for a principal component analysis of 24 log-transformed measurements of *T. pachychilus* and *T. microstoma* (n = 17). *Trematocranus microstoma*: white triangle; *Trematocranus pachychilus* holotype: white circle; paratypes: grey circle.

**Figure 2.**
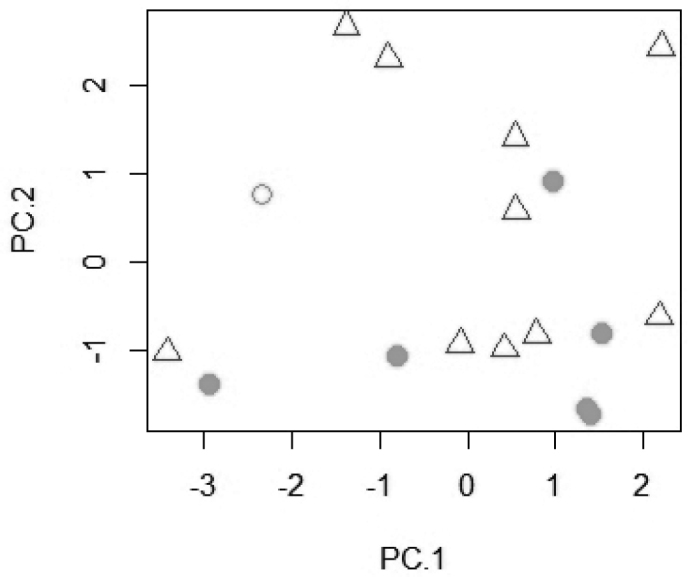
Scatter plot of PC 1 against PC 2 for a principal component analysis of eleven counts of *T. pachychilus* and *T. microstoma* (n = 17). *Trematocranus microstoma*: white triangle; *Trematocranus pachychilus* holotype: white circle; paratypes: grey circle.

**Figure 3.**
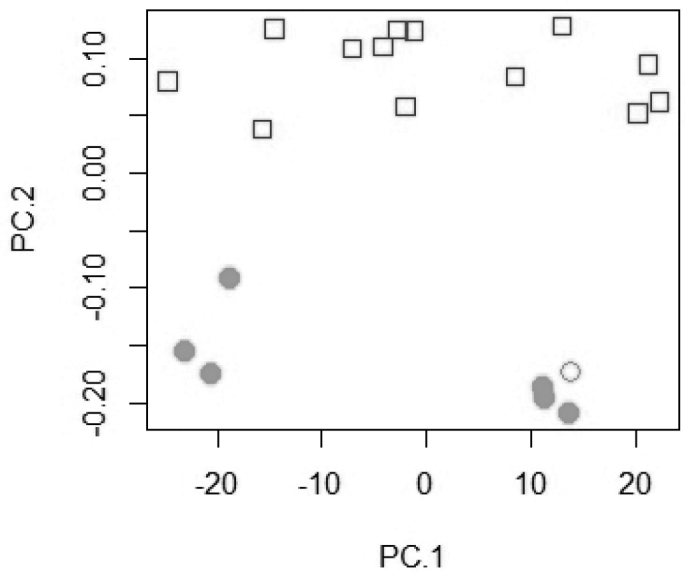
Scatter plot of PC 1 against PC 2 for a principal component analysis of 24 log-transformed measurements of *T. pachychilus* and *T. placodon* (n = 20). *Trematocranus placodon*: white square; *Trematocranus pachychilus* holotype: white circle; paratypes: grey circle.

**Figure 4.**
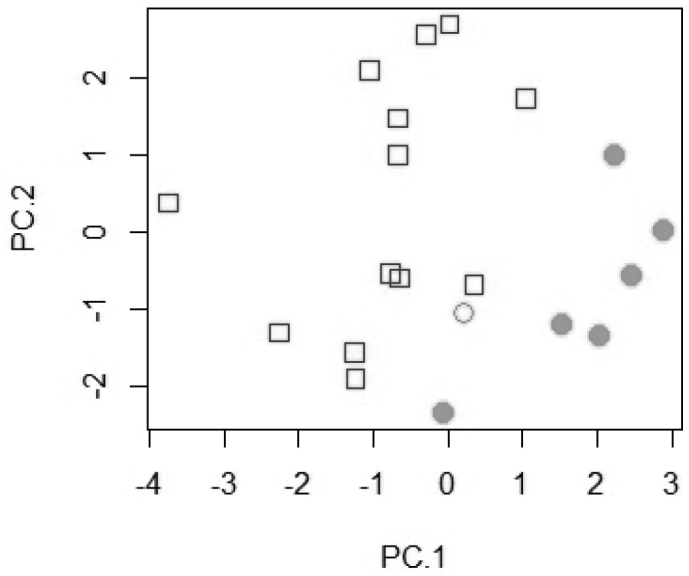
Scatter plot of PC 1 against PC 2 for a principal component analysis of 11 counts of *T. pachychilus* and *T. placodon* (n=20). *Trematocranus placodon*: white square; *Trematocranus pachychilus* holotype: white circle; paratypes: grey circle.

A PCA on the log-transformed measurements including *T. pachychilus* and *T. microstoma* resulted in a separation on the third principal axis (Fig. 1). The most important loadings on PC 3 are of the lachrymal depth, snout length, cheek depth, and the width of the dentigerous area of the lower pharyngeal bone. In a PCA on the raw meristics, *T. pachychilus* and *T. microstoma* could not be separated (Fig. 2). The most important loadings on PC 1 are for the number of longitudinal scales, upper and lower lateral line scales, and dorsal fin spines. On PC 2 the highest values are for the number of pharyngeal tooth rows, the upper and lower gill rakers, and the dorsal fin spines. A PCA on 24 log-transformed measurements including *T. pachychilus* and *T. placodon* resulted in a clear separation on the second principal axis of the two species (Fig. 3). The most important loadings on PC 2 are for measurements of the pharyngeal bone (depth, width, and length) and its dentigerous area. In a PCA on the raw meristics, *T. pachychilus* and *T. placodon* separate with some overlap mostly on PC 1 (Fig. 4). The most important loadings on PC 1 are for the numbers of scales on the upper lateral line and the longitudinal line, upper jaw teeth, and pharyngeal teeth rows. On PC 2 the highest values are for the number of dorsal fin rays, scales on the lower lateral line, dorsal fin spines, and pharyngeal teeth rows.

#### Trematocranus pachychilus sp. n.

Taxon classificationAnimaliaPerciformesCichlidae

http://zoobank.org/50FF20F1-74E0-4C11-90AC-37FAB2121378

##### Type material.

Holotype: MRAC 99-041-P-4781, 154.6 mm SL, Jafua Bay, Lake Malawi, Mozambique, 12°07.13'S, 34°45.89'E, Coll. Snoeks & Hanssens (4 April 1998). Paratypes: MRAC 99-041-P-4782, 151.9 mm SL, same data as holotype; MRAC 99-041-P-5037-5041 (5), 117.7 – 154.5 mm SL, same data as holotype.

##### Differential diagnosis.

*Trematocranus pachychilus* is clearly distinct from all other known species of the genus by its thick lips. It can further be distinguished from *T. labifer* by its molariform pharyngeal dentition while the latter has slender teeth on the lower pharyngeal jaw (Eccles and Trewavas 1989).

**Figure 5.**
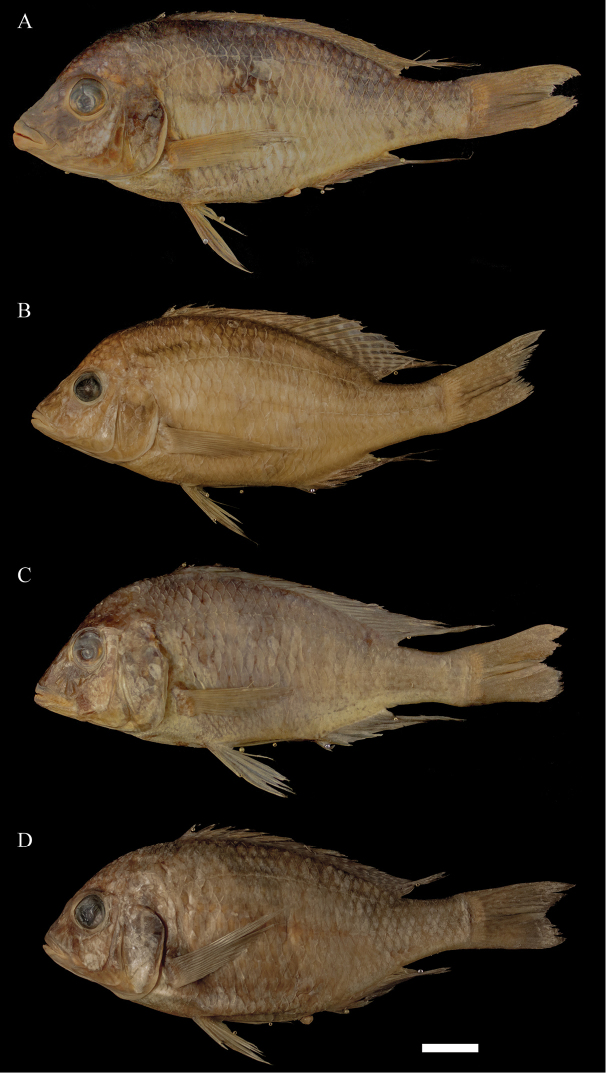
Photographs of preserved specimens: **A** *Trematocranus pachychilus*, holotype, MRAC 99-041-P-4781, adult male, 154.6 mm SL, Lake Malawi, Jafua Bay **B** *T. microstoma*, MRAC 99-041-P-4787, 143.7 mm SL, Lake Malawi, Mazinzi Bay **C** *T. placodon*, MRAC 99-041-P-4776, 153.8 mm SL, Lake Malawi, Jafua Bay **D** *T. placodon*, MRAC 99-041-P-4798, 149.3 mm SL, Lake Malawi, Senga Bay. Scale bar: 2 cm.

In *T. placodon* (Fig. 5C, D) lips are usually very small. While *T. pachychilus* has a rather concave head, as in *T. microstoma*, *T. placodon* has a more distinct convex head. In both *T. pachychilus* and *T. placodon*, the smaller specimens have a straighter head profile. Coincidentally, in two large specimens of *T. placodon*, found at Jafua Bay, the head is slightly concave. The teeth of *T. pachychilus* are less broad, more densely spaced and slightly less numerous (36–47 vs. 41–57) than in *T. placodon*. *Trematocranus pachychilus* has more upper lateral line scales (22–24 vs. 19–22) and more dorsal fin rays (11–12 vs. 9–11) than *T. placodon*. The pharyngeal bones of *T. pachychilus* are less developed than in *T. placodon*. They are shallower (9.4–10.4% HL vs 13.8–16.8% HL), narrower (29.7–33.2% HL vs. 36.1–48.7% HL) and shorter (21.8–30.8% HL vs. 29.8–35.9% HL) than those of *T. placodon*.

The dentigerous area is also narrower than in *T. placodon* (21.9–24.4% HL vs. 24.2–27.5% HL). Both species have large, molariform pharyngeal teeth, although in *T. pachychilus* the lateral teeth are smaller and more numerous than in *T. placodon*. The number of teeth on the posterior pharyngeal row in *T. pachychilus* is higher than in *T. placodon* (23–27 vs.11–23).

**Figure 6.**
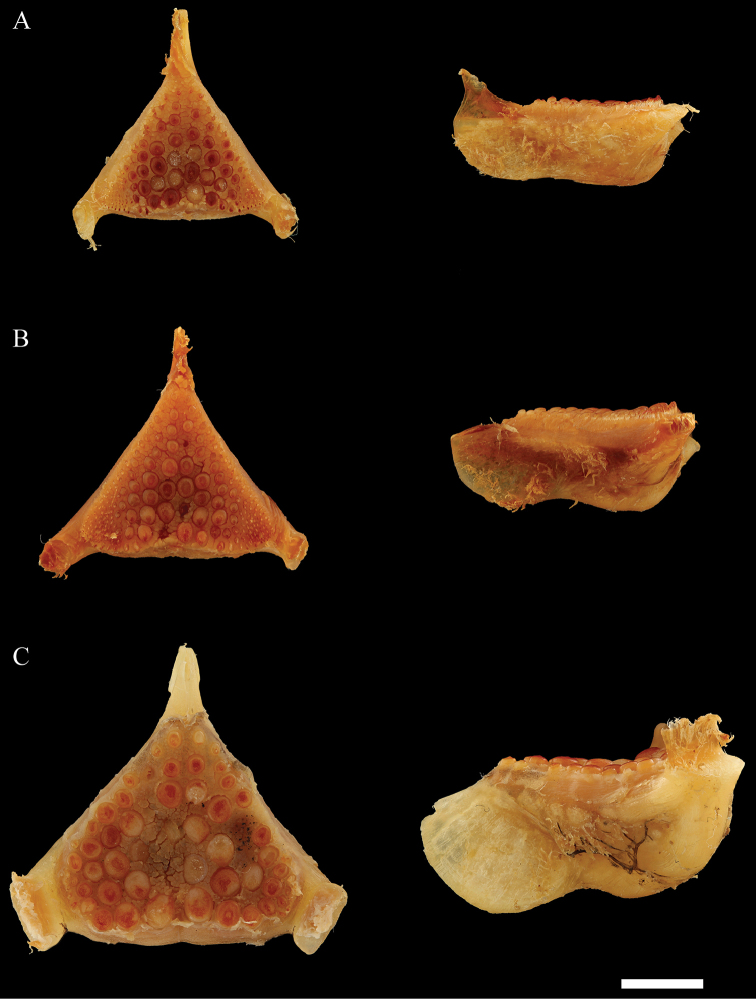
Lower pharyngeal bones, dorsal view (left) and lateral view (right): A. *Trematocranus pachychilus*, paratype, MRAC 99-041-P-4782, 151.9 mm SL, Lake Malawi, Jafua Bay; B. *T. microstoma*, MRAC 99-041-P-4787, 143.7 mm SL; C. *T. placodon*, MRAC 99-041-P-4776, 153.8 mm SL. Scale bar: 5 mm.

In *T. microstoma* (Fig. 5B) the lips are intermediate except for some specimens of Mazinzi Bay (see discussion). The snout of *T. pachychilus* is shorter than that of *T. microstoma* (34.8–40.9% HL vs. 38.0–42.4% HL). *Trematocranus pachychilus* has bicuspid oral teeth on the outer rows while *T. microstoma* has slender unicuspid teeth. It has fewer inner teeth rows (3–5 vs. 6–8) on the lower jaw than *T. microstoma*. The lachrymal depth is clearly shorter than in *T. microstoma* (22.9–29.7% HL vs. 29.3–32.0% HL), while the eye diameter is larger (25.2–29.5% HL vs. 21.2–25.5% HL). The pharyngeal bones (Fig. 6) are more developed in *T. pachychilus* than in *T. microstoma*. The dentigerous area of *T. pachychilus* is wider than in *T. microstoma* (21.9–24.4% HL vs. 18.8–23.6% HL). The dentition of the lower pharyngeal bone is similar in both species, with the median teeth enlarged and the lateral teeth small and numerous.

##### Description.

Based on the holotype and six paratypes examined. See Figures 5A and 6A. Qualitative observations are made in the context of Lake Malawi haplochromine cichlids as was done by Snoeks (2004).

Body deep and laterally compressed. Head steep. Most specimens with a slight concavity at eye level; some, especially smaller specimens, with a merely straight head profile. Snout pointed. Mouth terminal. Lips very thick and equally developed in most specimens. Larger specimens with thicker lips than small specimens; smaller specimens often with a clear boundary between lower lip and chin. Maxilla does not extend to level of eye.

Teeth not readily observable, being to a large extent or fully covered by the fleshy gums. Outer row of teeth on upper and lower jaws with mainly unequally bicuspid and some unicuspid teeth in the postero-lateral parts; teeth slender, generally straight and slightly curved inwards; crown slightly wider than base. Anterior bicuspid teeth more pointed and sharp; lateral bicuspid teeth often rounded. Inner rows mostly with unicuspid teeth. Larger specimens with more bicuspid teeth anteriorly in inner rows. Inner teeth rows difficult to observe; 3–4 inner rows on upper jaw (counted in four specimens); 3–5 on lower jaw (six specimens).

Pectoral fins implanted slightly behind level of dorsal fin origin. Pelvic fin origin positioned slightly more backwards. Anal fin implanted anterior to level of first soft dorsal fin ray.

Lower pharyngeal bone triangular, large, wider than long, and deep. Teeth molariform; central teeth much larger than lateral teeth. Outermost teeth on the posterior rows very small and numerous.

##### Colour pattern in preservation.

Body generally brown; some specimens slightly more greyish. Dorsum darker than belly. Very dark-coloured on the dorsal parts of head and body contiguous with dorsal fin base in most specimens. Some large darker spots usually on operculum, supra-pectoral in front of dorsal fin origin, dorso-lateral above and on upper lateral line in middle of flank, supra-anal near end of upper lateral line, and caudal peduncle. Clear dark maculae on spiny part of dorsal fin; sometimes also on soft dorsal fin part and caudal fin. Pelvic and anal fins yellowish brown in females; fin base and distal part clearly darker in males. One specimen with a vague brown midlateral stripe along flank.

##### Etymology.

The specific name, *pachychilus*, means thick-lip and is derived from the Greek words παχυς (fat, adipose, plump) and χειλος (lip, edge) with reference to the diagnostic character, the thick lips.

##### Geographical distribution.

The specimens of *T. pachychilus* were only found at Jafua Bay, north of Cobue in Lake Malawi, Mozambique. All specimens were caught in a single haul of a bottom trawl at a depth of 10.8–12.8 m (12°07.13'S, 34°45.89'E).

##### Ecology.

Shell fragments of snails were observed in the stomachs of some specimens on X-rays. Stomach analyses were not performed so as to not damage the type series. Thick and fleshy lips are associated with rocky habitats in cichlids, since fish feeding between rocks use their lips as a seal to be able to suck out their prey from crevices and as a protection against sharp rocks (Machado-Schiaffino et al. 2014). Since all specimens were found in bottom trawls over soft substrates, we are unsure about the association of the new species with rocky habitats.

### Key to the species of *Trematocranus*

**Table:** 

1	Slender and sharp pharyngeal teeth	***T. labifer***
–	Molariform pharyngeal teeth	**2**
2	Unicuspid oral teeth on outer row; large lachrymal depth (29.3–32.0% HL); narrow lower pharyngeal bone (19.1–30.9% HL)	***T. microstoma***
–	Bicuspid oral teeth on outer row; small lachrymal depth (22.9–29.7% HL); wide lower pharyngeal bone (29.7–48.7% HL)	**3**
3	Oral teeth widely spaced, broad; 41–57 oral teeth on outer row of upper jaw; 11–23 lower pharyngeal teeth rows; pharyngeal bone massively enlarged; lips small; 19–22 upper lateral line scales	***T. placodon***
–	Oral teeth densely spaced, slender; 36–47 oral teeth on outer row of upper jaw; 23–27 lower pharyngeal teeth rows; pharyngeal bone enlarged; thick lips; 22–24 upper lateral line scales	***T. pachychilus* sp. n.**

## Discussion

According to the revised classification by Eccles and Trewavas (1989), there were three valid species in the genus *Trematocranus*. Fish caught at Jafua Bay, Lake Malawi, by Snoeks and Hanssens in 1998 are found here to be a new species, *Trematocranus pachychilus* sp. n.

*Trematocranus pachychilus* is distinguished from all other congeners by its thickened and fleshy lips. *Trematocranus labifer* is known to have slender teeth on the pharyngeal jaws, while all other *Trematocranus* species have a molariform dentition (Eccles and Trewavas 1989, Snoeks and Hanssens 2004). Therefore, it was not included in the detailed morphometric analyses. In these analyses, it was shown that the new species differs in several characters from *T. microstoma* and *T. placodon*. *T. pachychilus* has thick lips and bicuspid oral teeth on the external row, while in *T. microstoma* lips are generally less thick and the outer oral jaw teeth are unicuspid. In addition, there are some differences in measurements, but there appears to be no clear separation between *T. pachychilus* and *T. microstoma* based on meristic data (Table 1, Fig. 2). They do differ in head shape. *Trematocranus pachychilus* can be distinguished from *T. placodon* by its thick lips and less hypertrophied pharyngeal bone, oral and pharyngeal dentition, and in a few measurements and meristics (Table 1, Figs 3–4).

While all specimens of *T. pachychilus* have hypertrophied lips, some variability in this characters in the two other species was observed. Two specimens of *T. placodon* were a bit aberrant. They clearly have the massive pharyngeal bones typical for *T. placodon* (Fig. 6C) and, in the analyses of the measurements and meristics, are associated with the other members of this species (Figs 3–4). However, one specimen (MRAC 99-041-P-4779) has both the upper and lower lips thickened, while the other specimen (MRAC 99-041-P-4780) only has a slightly thicker upper lip. Three *T. microstoma* specimens also show enlarged lips (MRAC 99-041-P-4789-4791) that are thicker than in their conspecifics. At present, we have difficulties in interpreting the variability in lip morphology in both species. Genetically determined intraspecific variability may be large in these species, but on the other hand, lips are also known to be morphologically plastic in cichlids. In an experimental study on Neotropical cichlids, both a genetic and a phenotypic plastic factor was found to be involved in the development of thick lips. (Machado-Schiaffino et al. 2014). Whether or not this is also the case in Lake Malawi and therefore explains the case of the two specimens of *T. placodon* and the three of *T. microstoma* is unclear. Alternatively, the hypothesis of hybridization cannot be excluded either. However, it should be stressed that except for lip thickness, the two specimens of *T. placodon* and the three of *T. microstoma* display the diagnostic characters typical for their respective species.

Pharyngeal jaw morphology has also been reported as phenotypically plastic. For instance, by feeding on hard food items, the pharyngeal jaws of cichlids have been reported to become larger with a more developed horn and keel and their teeth more numerous and stouter during ontogeny (Greenwood 1965, Hoogerhoud 1986, Gunther et al. 2013). However, in the case of *Trematocranus*, we see a clear distinction in pharyngeal jaw morphology and its dentition between the species, supported by other morphological features and therefore have no indication of such plasticity in the populations examined. In addition, significant differences in lower pharyngeal jaw morphology due to phenotypic plasticity have only been obtained by a highly differential feeding regime (soft versus hard items) in an experimental setup (Hoogerhoud 1986, Gunther et al. 2013), while intraspecific differences in nature have been found only in one African cichlid *Astatoreochromis alluaudi* in allopatric populations living in different environments and probably linked to the presence/absence of snails (Greenwood 1965). Such diverging (experimental and natural) conditions do not seem to correspond to the conditions in which *T. pachychilus* and *T. placodon* were found, both in trawls over soft bottoms far remote from rocks in order not to damage the net, and in the presence of snails (see below).

*Trematocranus pachychilus* and *T. placodon* are both found at Jafua Bay. Because of the obvious differences in morphology we expect their trophic niche to be different. Nevertheless, both appear to feed on molluscs. *Trematocranus placodon* has been reported to eat molluscs (Mtethiwa et al. 2015). Indeed, some fragments of bivalve shells and snails were found in the pharyngeal area of specimens of this species, while dissecting the pharyngeal bones. Shell fragments of snails were observed in the stomachs of some specimens of both *T. placodon* and *T. pachychilus* on X-rays. It would be very interesting to see if *T. pachychilus* also lives in other bays across the lake, and whether they live sympatrically in other areas with *T. placodon*. If more specimens and data become available, a more in-depth study could relate the morphological differences found with their trophic ecology.

## Conclusions

Morphometric analyses confirm that *T. pachychilus* is a new species within the genus *Trematocranus*, next to the three valid species *T. microstoma*, *T. labifer* and *T. placodon*. Found at Jafua Bay of Lake Malawi, it co-occurs with the latter. It was compared with *T. microstoma* and *T. placodon* using a principal component analysis on measurements and counts. The most striking characteristics to separate the four species are the dimensions of the pharyngeal bone, the number of pharyngeal teeth rows, pharyngeal dentition, oral dentition, and lip thickness. More data on the distribution and morphological variability are needed to resolve some questions regarding the sympatry of *T. placodon* and *T. pachychilus* sp. n.

## Comparative material

*Trematocranus placodon*: MRAC 99-041-P-4776-4778 (3), 125.1–153.8 mm SL, Jafua Bay, Lake Malawi, Mozambique, 12°07.13'S, 34°45.89'E, 4 April 1998; MRAC 99-041-P-4779-4780 (2), 161.1–163.1 mm SL, Jafua Bay, Lake Malawi, Mozambique, 12°07.13'S, 34°45.89'E, 4 April 1998; MRAC 99-041-P-4798-4806 (6 of 9), 116.0–149.3 mm SL, Senga Bay, Lake Malawi, Malawi, 13°46.73'S, 34°37.91'E, 21 September 1997; MRAC 6539-6540 (2), 138.1–162.0 mm SL, Senga Bay, Malawi, Lake Malawi, 13°46.73'S, 34°37.91'E, 21 September 1997.

*Trematocranus microstoma*: MRAC 99-041-P-4785-4786 (2), 166.7–182.7 mm SL, Kanda Bay, Lake Malawi, Malawi, 11°56.84'S, 34°08.28'E, 3 June 1997; MRAC 99-041-P-4787-4788 (2), 143.7–154.9 mm SL, Mazinzi Bay, Lake Malawi, Malawi, 14°09.60'S, 34°58.72'E, 11 October 1997; MRAC 99-041-P-4789-4791 (3), 135.4–158.2 mm SL, Mazinzi Bay, Lake Malawi, Malawi, 14°05.33'S, 35°01.94'E, 11 October 1997; MRAC 99-041-P-4792-4797 (3 of 6), 126.7–144.9 mm SL, Chembe Bay, Lake Malawi, Malawi, 14°01.57'S, 34°49.88'E, 27 May 1997.

## Supplementary Material

XML Treatment for Trematocranus pachychilus
